# Genome Sequences of *Gordonia* Phages Hotorobo, Woes, and Monty

**DOI:** 10.1128/genomeA.00598-16

**Published:** 2016-08-11

**Authors:** Welkin H. Pope, Jameson P. Davis, Sirinya O’Shea, Anastasia C. Pfeiffer, Alexandra N. Rich, Jason C. Xue, Kathleen A. Shedlock, Ann-Catherine J. Stanton, Emily C. Furbee, Sarah R. Grubb, Marcie H. Warner, Matthew T. Montgomery, Rebecca A. Garlena, Daniel A. Russell, Deborah Jacobs-Sera, Graham F. Hatfull

**Affiliations:** Department of Biological Sciences, University of Pittsburgh, Pittsburgh, Pennsylvania, USA

## Abstract

Hotorobo, Woes, and Monty are newly isolated bacteriophages of *Gordonia terrae* 3612. The three phages are related, and their genomes are similarly sized (76,972 bp, 73,752 bp, and 75,680 bp for Hotorobo, Woes, and Monty, respectively) and organized. They have extremely long tails and among the longest tape measure protein genes described to date.

## GENOME ANNOUNCEMENT

The genetic diversity of phages infecting *Gordonia* spp. is not well defined, and relatively few *Gordonia* phage genome sequences have been reported (1–5). The Science Education Alliance Phage Hunters Advancing Genomics and Evolutionary Science (SEA-PHAGES) program provides an opportunity to isolate and characterize *Gordonia* phages and to compare their diversity with the large collection of mycobacteriophages (6, 7).

Three phages were isolated from soil samples collected in Pittsburgh, Pennsylvania, USA, using *Gordonia terrae* 3612 as a host: Monty and Woes by direct plating, and Hotorobo by enrichment on *G. terrae* 3612. Phages were plaque purified and amplified; dsDNA was extracted and sequenced using an Illumina MiSeq. Single-end 140-bp reads were assembled using Newbler into a single major contig for each phage with 272-fold, 637-fold, and 437-fold coverage and genome sizes of 73,752 bp, 75,680 bp, and 76,792 bp for Woes, Monty, and Hotorobo, respectively. Each has 192-bp direct terminal repeats. The Hotorobo and Monty genomes are very closely related and share 98% average nucleotide identity, spanning 96% of their genome lengths. Woes is more distantly related and shares 79% nucleotide identity with Hotorobo, spanning 69% of its genome length. The three phages share nucleotide sequence similarity with *Gordonia* phages GTE7 and GMA7 but at only 71 to 72% nucleotide identity and spanning only 46 to 51% of their genome lengths.

Genes were predicted using DNA Master (http://cobamide2.bio.pitt.edu), Glimmer (8), GeneMark (9), Phamerator (10), Aragorn (11), and tRNAscan-SE (12), resulting in 108, 105, and 91 protein-coding genes in Hotorobo, Monty, and Woes, respectively, and they each have a single tRNA^Asn^ gene that is unusually located between the major tail subunit gene and the tail assembly chaperone genes. Functional gene assignments were made using BLAST (13) and HHPred (14).

Hotorobo, Monty, and Woes have siphoviral morphologies with isometric heads and extremely long tails approximately 540 nm in length. These are among the longest phage tails reported, and the genomes have corresponding long tape measure protein genes, as expected. The tape measure protein genes in Hotorobo and Monty are 9,984 bp, and 9,969 bp in Woes, accounting for over 13% of the coding capacity of the entire genomes. These are slightly longer than the tape measure protein genes in GMA7 and GTE7, both of which are 9,141 bp long.

The lysis genes in Hotorobo, Monty, and Woes are located downstream of the tail genes, and the muramidase and peptide functions are encoded in separate genes, adjacent to a putative holin gene. All three genomes encode a putative lysin esterase, but it is displaced approximately 5 kb from the other lysis genes. The phages do not encode recognizable integrase or repressor genes and are unlikely to be temperate.

### Accession number(s).

The genomes of Hotorobo, Monty, and Woes are available from GenBank under the accession numbers KU963245, KU998241, and KU998240, respectively.
